# Glucocorticoid Negative Feedback in Regulation of the Hypothalamic-Pituitary-Adrenal Axis in Rhesus Monkeys With Various Types of Adaptive Behavior: Individual and Age-Related Differences

**DOI:** 10.3389/fendo.2019.00024

**Published:** 2019-02-13

**Authors:** Nadezhda Goncharova, Olga Chigarova, Natalia Rudenko, Tamara Oganyan

**Affiliations:** Laboratory of Experimental Endocrinology, Research Institute of Medical Primatology, Sochi, Russia

**Keywords:** hypothalamic-pituitary-adrenal axis, glucocorticoid negative feedback, mineralcorticoid receptor agonist, glucocorticoid receptor agonist, individual differences, behavior, aging, rhesus monkeys

## Abstract

The study of the mechanisms underlying the increased vulnerability of the individual to stressful environmental factors in different age periods is of great relevance for prevention and effective treatment of stress-dependent diseases that are widespread in the population of aging individuals. The purpose of our study was to investigate the individual and age-related features of the glucocorticoid negative feedback in regulation of the hypothalamic-pituitary-adrenal (HPA) axis, the key adaptive neuroendocrine system, in experiments with physically healthy young and old female rhesus monkeys with administration of mineracorticoid receptor (fludrocortisone) and glucocorticoid receptor (dexamethasone) agonists. We studied the monkeys with increased trait anxiety and depression-like behavior (DAB) characterized, as previously was shown, by the increased vulnerability to acute stress and the animals with normal standard behavior (SB) as the control. The pronounced individual differences in the reaction of HPA axis to fludrocortisone and dexamethasone in young animals were found. Young animals with DAB showed a lower sensitivity of HPA axis to the inhibitory effect of both fludrocortisone and dexamethasone compared with young animals with SB. At the same time, there were no significant intergroup differences in the concentration of ACTH and cortisol in response to placebo injection, i.e., in basal conditions. The old individuals with DAB demonstrated the essential relative resistance of HPA axis to fludrocortisone test and higher basal plasma levels of cortisol and ACTH in the evening (the period of HPA axis low circadian activity) compared to old SB animals. In the same time, the intergroup differences in the response of HPA axis to dexamethasone administration were leveled due to age-related increase in sensitivity of HPA axis to dexamethasone in animals with DAB. These data testify the pronounced intergroup and age differences in the feedback regulation of HPA axis, presumably resulting from unequal individual, and age-related changes in the activity of mineralcorticoid and glucocorticoid receptors in the brain structures supporting the functions of HPA axis. The maximum age disorders in functioning of the negative feedback mechanism in the regulation of HPA axis are characteristic of animals with DAB, which, apparently, underlie the increased vulnerability of these animals to stress exposure.

## Introduction

Expansion of the stress factors range (terrorism, environmental ill-being, local wars, etc.) determines relevance of the problem of stress impact on human health. Changes in the demographic situation with the increase in the proportion of older people and the emergence of a number of so-called age-related diseases in which stress plays an important role, in young and middle-aged people make it important the search for mechanisms and biomarkers to identify individuals with an increased vulnerability to stress in different age periods. The probability of the post-stress syndrome onset is sharply increased by the age (1–6). However, the same stressful event can cause various symptoms/severity or even lack of symptoms of stress-related disorder in different individuals, including the elderly that is associated with features of the higher nervous activity (7–15). For example, a number of studies indicate a higher stress reactivity of the HPA axis, the key adaptive neuroendocrine system, in healthy men with increased anxiety (7) and in older age groups with depression (11). Hyperactivation of the HPA axis was detected in rodents with depression-like and anxiety-like behavior (8, 9), as well as in persons with depression (10, 16). Differences in the stress response of the HPA axis were observed in immature nonhuman primates with high and low trait anxiety (17) and in old female rhesus monkeys with depression-like and anxiety-like behavior, on the one hand, and usual standard behavior, on the other hand (13, 14). Thus, old animals with depression-like and anxiety-like behavior were most vulnerable to negative aspects of acute stress exposure and showed the greatest disturbances in the functioning of the HPA axis accompanied by an increase in the concentration of biomarkers of aging (13, 14). Significant differences in the functioning of the HPA axis were also demonstrated by young mature animals with depression-like and anxiety-like behavior (18–20). In these animals, an increased ACTH response to repeated stress exposure and also to functional tests with arginine vasopressin (AVP) and corticotrophin releasing hormone (CRH) was revealed compared to young females with standard behavior (18–20).

What are the mechanisms of differences in the stress of HPA axis reactivity in individuals with standard behavior and depression-like and anxiety-like behavior revealed by us and other authors? Earlier, we obtained data to conclude that in old primates with depression-like and anxiety-like behavior the activity of specific vasopressin V1b receptors on corticotrophs of the anterior pituitary was increased. So, we supposed an increase in the activity of specific receptors to CRH (CRHRI) on corticotrophs of the pituitary gland (13, 18, 19, 21). In turn, an increase in the concentration of specific receptors on corticotrophs in individuals with depression-like and anxiety-like behavior could be a consequence of increased synthesis and secretion of CRH and AVP in the pituitary portal system. Increased neurohormone production can be caused by disturbances in the HPA axis activity regulation by the glucocorticoid negative feedback, the most important mechanism of HPA axis regulation.

The central sensors of glucocorticoid negative feedback in regulation of the HPA axis are 2 glucocorticoid receptors: high affinity mineralcorticoid receptor (or type 1 receptor, MR) and low affinity glucocorticoid receptor (or type 2 receptor, GR) expressed in specific brain structures. Both glucocorticoid receptors mediate effect of glucocorticoid hormones on the brain, acting in the cell nuclei as activators of gene transcription factors. MR is predominantly expressed in the limbic system, mainly in the hippocampus and also the medial prefrontal cortex and some other areas of the limbic system (4, 22, 23), where it co-localizes with GR, which binds corticosteroid with a 10-fold lower affinity. The functional role of MR is proposed to be proceeded through tonic inhibitory projections, mediated by c-aminobutyric acid (GABA)ergic neurons, to the paraventricular nuclei (PVN) of the hypothalamus (4, 23, 24). Hippocampal MR involved in the maintenance of the basal HPA activity, mainly at the nadir of the circadian rhythm, when hippocampal MR are significantly occupied (24, 25). GR, in turn, are widely expressed throughout the central nervous system, but mostly in hypothalamic neurons and corticotroph cells in the pituitary gland (4, 25). GR play an important role in the control of HPA axis on the negative feedback mechanism, when the level of circulating glucocorticoids is high, for example, after stress or circadian peak (25). Recently, rapid non-genomic effects have been demonstrated for MR and GR (26).

Disorders of the feedback mechanism in HPA axis regulation and associated hypercortisolism are characteristic of aging processes (4, 24), neurocognitive disorders, psychiatric, and neurodegenerative diseases (16, 24, 27–29). The pronounced correlation of disturbances in the glucorticoid negative feedback with psychiatric diseases and neurocognitive deficits takes place mainly because MR and GR not only participate in the regulation of HPA axis activity but also control cognitive processes. For example, GR facilitates memory consolidation by processing and storing experience (30, 31). MR are involved in evaluating a new condition, behavioral flexibility, selective attention, and emotional behavior (32, 33).

There are many publications that testify to the pathophysiological role of disturbances in the negative feedback regulation of the HPA axis in the progression of aging processes and mental disorders. In the same time, there is practically no information on the features of the functioning of this mechanism in healthy individuals differing in the features of higher nervous activity (in particular, peculiarities of behavior in mild/moderate stress conditions). Previously we proposed a natural animal model (female rhesus monkeys with standard, aggressive, depression-like, and anxiety-like behavior) to study individual differences in the functioning of HPA axis (12, 18). In addition, as mentioned above, we studied the intergroup and age-related features of HPA axis response to acute psycho emotional stress exposure in monkeys with standard, depression-like, and anxiety-like behavior (13, 14). These research revealed pronounced individual and age differences in the stress responsiveness of HPA axis.

The purpose of the present study was to investigate the individual and age-related features of the glucocorticoid negative feedback in regulation of HPA axis on the model of rhesus monkey females with control standard behavior and with depression-like and anxiety-like behavior using functional tests with agonists of MR (fludrocortisone) and GR (dexamethasone). We demonstrated for the first time that the maximum age disorders in the functioning of the negative feedback in the regulation of HPA axis with a pronounced decreased sensitivity to fludrocortisone and increased evening time HPA axis activity in the basal conditions are characteristic of animals with DAB. These disturbances, apparently, underlie the increased vulnerability of these animals to the acute stress that we identified earlier.

## Materials and Methods

### Animals

#### The Conditions of Keeping Monkeys in the Nursery

Eighteen young adult (5–8 years) and 19 old (21–30 years) healthy female rhesus monkeys (*Macaca mulatta*) were used in the experiments. The monkeys originated from the Adler monkey colony (Research Institute of Medical Primatology, Sochi, Russia). The animals usually were housed in open enclosures (size 250 m^2^ × 5 m and 650 m^2^ × 5 m, housing 10–15, and 40–50 individuals of various age, including newborns and elderly animals, respectively) or cages designed for group housing (size 10 m^2^ × 2.75 m, housing 3–5 individuals). Lighting, humidity, and temperature were as per the ambient environment, though each enclosure featured small closed sections, which are heated in winter, where animals can hide in adverse weather conditions.

During the observation period the animals were moved into individual metabolic cages (size 80 × 80 × 80 cm) in a separate room with narrow windows, controlled temperature, and natural illumination. Temperature varied from 26 to 28°C, taking into account the time of day. The lighting was daylight approximately from 06.00 to 18.00. Additional artificial illumination could be switched on as required, for example when taking blood samples in the evening, in which case soft illumination was switched on for 15–20 min. All experiments were carried out in the period of June-August when ovarian cycles are not typical for female rhesus monkeys. The animals were fed pellets prepared in the Institute according to the technique of Altromin Spezialfutter GmbH & Co. KG (Lage, Germany). The pellet diet was complemented with bread, boiled eggs, and fresh vegetables and fruit. Water was available *ad libitum*.

### Health Assessment

The state of health of the animals was monitored by noninvasive methods (assessment of mobility, condition of a hair cover, condition of stool and urine, microbiological evaluation of rectal smears, surveying for signs of ovarian cycles—color, and a degree of swelling of a “sexual skin”) and also with use of biochemical analysis of blood and blood counts (34).

### Preparing Monkeys for Experiments

Before the experiments, the animals were adapted to living in metabolic cages and to the procedure of bleeding for 4 weeks. During this period the animals were attended by the same keepers and researchers. The animals were subjected to blood sampling followed by food reinforcement (fruit, sweets) once or twice weekly. It was established previously that this period of time is sufficient for elimination of orientation and aggressive-defensive unconditional reflexes of animals to a new habitat as well as to experimental procedures (so called “procedural” stress). It should be noted that we continued to reward animals with food reinforcement after each blood sampling procedure throughout all experiment in order to attempt to eliminate any defensive reflex and preventions of development of procedural stress.

### The Experimental Groups of Monkeys, Assessment of Their Behavior

The animals‘ behavior was recorded while they were housed in the metabolic cages, both during the period of adaptation, and throughout experimentation. Classification of behavior was done according to recommendations for laboratory primates (35, 36) and as described earlier (12, 14). Depending on behavioral features, both young and old animals were divided into two groups: the first groups comprised 9 young adult (6.6 ± 0.46 years, 5.1 ± 0.25 kg) and 11 old (25.8 ± 0.96 years, 5.6 ± 0.31 kg) animals with healthy active adaptive behavior (standard behavior, SB). The second groups consisted of 9 young adult (6.4 ± 0.39 years, 5.0 ± 0.19 kg) and 8 old (25.0 ± 0.79 years, 6.4 ± 0.58 kg) animals with maladaptive depression-like and anxiety-like behavior (DAB). Division of the monkeys into SB (control) and DAB was done according to their behavior in response to appearance of the experimenter in the room, handling of the animals in order to prepare them for bleeding; further handling during the actual procedure of bleeding; food reinforcement immediately after the bleeding procedure. Division into these categories was distinctly accurate in 2–3 weeks of the adaptive period and remained same till the end of experiment which lasted on average 8 weeks, including the adaptation period. We did not use animals with unclear behavior characteristics.

The initial reactions of the animals in the first groups (SB) to experimenter (movements of the head and eyes in the direction of the stimulus, lying without movement) disappeared within the 2–4 weeks of the adaptation period. Following this period, these animals willingly came into a contact with researchers, were friendly, sat mainly toward the front of the cage, cooperated during blood sampling, and calmly accepted food reinforcements.

Animals with depression-like behavior assigned to DAB groups were characterized by a typical exaggerated avoidance of the experimenters, which persisted throughout the adaptation period, as well as through the rest of the experiment. The animals with anxiety-like behavior also included in the DAB groups showed unusually frantic motor activity and screeching during the bleeding procedure and did not respond to food reinforcement in the presence of experimenters.

We combined animals with depression-like and anxiety-like behavior in one group due to similarities in the character of the functioning of HPA axis at aging and under stress.

Analysis of the life history of the experimental animals revealed that two young animals with DAB (22%) were exposed to severe stress in early childhood (maternal deprivation due to maternal death in the period from 1 month to 9 months) and growing up separately from adult individuals, in the so-called “nursery” in individual cages until age 1.2 years, and then were kept in the cage designed for group housing, together with other immature animals, deprived of mother, and previously lived in the “nursery.”

### Methods

This study was based on a two-stage design. First, we studied glucocorticoid negative feedback in regulation of HPA axis on female rhesus monkeys using the test with fludrocortisone (FLUD, agonist MR) and then after 4 weeks we investigated negative feedback in regulation of HPA axis on the same monkeys using the test with dexamethasone (DEX, agonist GR).

### Test With Mineralcorticoid Receptor Agonist Fludrocortisone

In experiments with fludrocortisone, 9 young and 11 old animals were used with SB and 9 young and 8 old animals with DAB. The test with FLUD was carried out in 2 stages. First, all animals, regardless of age and behavior, were injected with saline (vehicle, placebo, 1 ml, intravenously) at 15.00. Blood sampling (no more than 1.0 ml) was performed prior to the injection of saline (0) and 3, 4, 6, 7, and 24 h after its administration, i.e., at 3, 6, 7, 9, 10, and at 3 p.m. of the next day.

Two days after the placebo administration, all the animals were injected at 15.00 h with a preparation of FLUD (Fludrocortisone acetate, MP Biomedicals, LLC., France), dissolved in physiological saline (0.5 mg/kg body weight, 1 ml, intravenously). Blood samples (not more than 1.0 ml) were taken in the same manner as in placebo, i.e., before administration of FLUD (0) and at 3, 4, 6, 7, and 24 h after its administration, i.e., at 3, 6, 7, 9, 10, and at 3 p.m. of the following day.

The levels of cortisol (CORT) were analyzed at each time point. In addition, the concentrations of adrenocorticotropic hormone (ACTH) were measured in the blood samples taken before the placebo and at various intervals after its administration. Test substances were administered in a single-blind fashion.

### Test With Glucocorticoid Receptor Agonist Dexamathasone

In the experiments with DEX we used 6 young and 7 old female rhesus monkeys with SB and 5 young and 6 old female rhesus monkeys with DAB. All animals received the drug DEX (CSPC Ouyi Pharmacutical Co., LTD, China), intramuscularly, at a dose of 0.5 mg/kg b. w.) at 09.00 h. Blood samples were taken prior to administration of DEX (0) and at 4, 6, 24, and 48 h after its administration.

The levels of CORT were analyzed at each time point. Test substances were administered in a single-blind fashion.

### Hormone Measurements

All blood samples were taken from cubital or femoral vein of the animals. Blood samples were collected in chilled tubes with EDTA (10.0 mg per 1 ml of blood) as the anticoagulant. At each time point 1.0 ml of blood was taken. Blood samples were immediately centrifuged at 2000 g at +4°C, plasma stored at −70°C for later analysis. Plasma levels of CORT and ACTH were measured by immunoenzyme assay using standard hormone kits (AlkorBio, Russia for total CORT, and Biomerica Inc., USA for ACTH). The sensitivity of the assay for CORT was 10.0 nmol/l. The intra-assay and inter-assay variation coefficients (C.V.) for CORT did not exceed 10 and 15%, respectively (mean C.V. for intra-assay = 7.4% and for inter-assay−11.0%). The sensitivity of the assay for ACTH was 0.22 pg/ml. The intra-assay and inter-assay variation coefficients for ACTH did not exceed 10 and 15%, respectively (mean C.V. for intra-assay = 7.4% and for inter-assay−10.0%).

This study was approved by the Ethical Committee of the Research Institute of Medical Primatology (Sochi). Animal caretaking was performed in accordance with the guidelines of the European Convention for the Protection of the Vertebrate Animals Used for Experimental and Other Scientific Purposes” (Strasbourg, 18.III.1986), Directive 2010/63/EU of the European Parliament and the Council of 22 September 2010 (on the protection of animals used for scientific purposes).

### Statistical Analysis

The experimental values are presented in tables and figures as means ± S.E.M. The statistical comparisons of the age and behavioral group differences were performed using one- and two-way analysis of variances (ANOVA) including *post hoc* Tukey‘s honest significant difference test for paired comparisons (Statistics 10 software package, Stat Soft. Inc., USA).

## Results

### Studies of the Glucocorticoid Negative Feedback in Regulation of HPA Axis in Young and Old Female Rhesus Monkeys With Various Types of Adaptive Behavior Using Test With Mineralcorticoid Receptor Agonist

#### Young Animals

**Figure 1:**
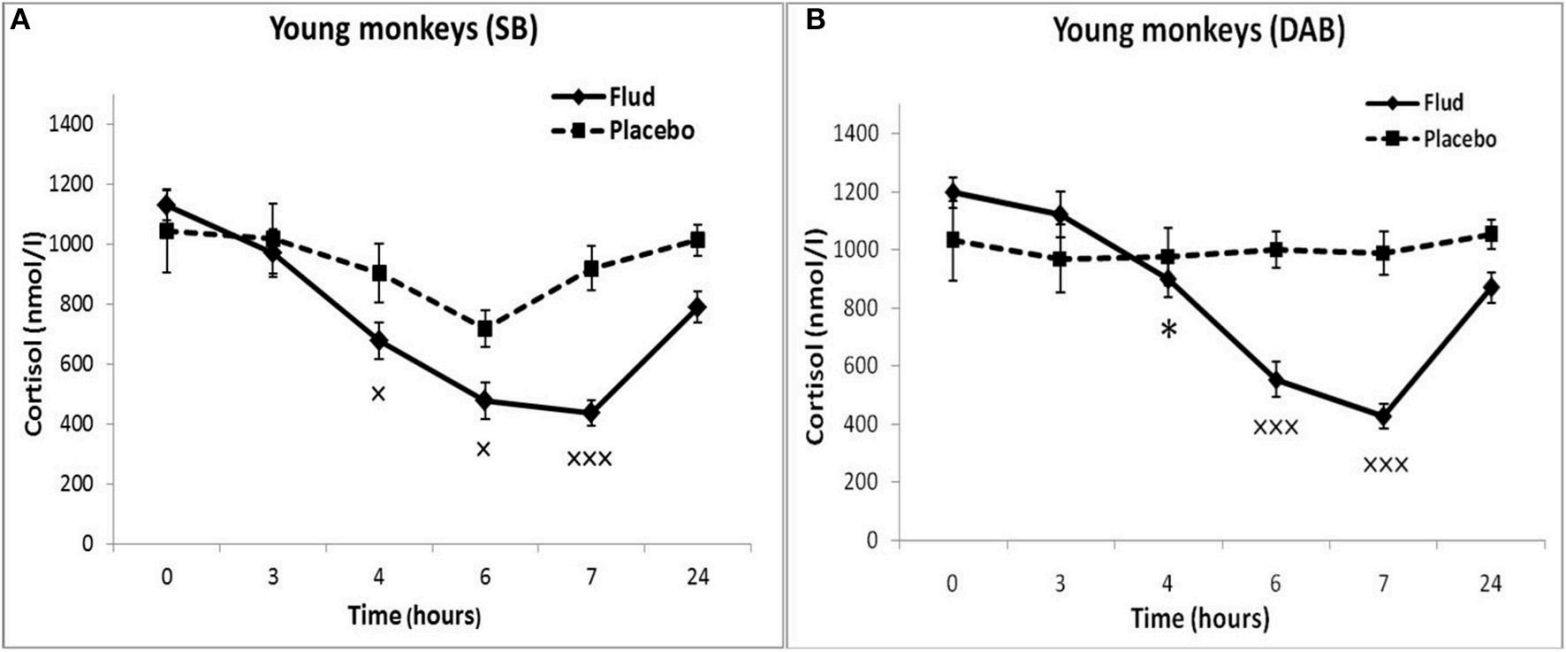
Dynamics of CORT concentration in peripheral blood plasma of young female rhesus monkeys with SB and DAB in response to administration of fludrocortisone and placebo (mean ± S.E.M). *p < 0.05 vs. relative values in animals with SB (*F* = 5.26; df = 1;15; *p* = 0.037). ×*p* < 0.05, ×××*p* < 0.001 vs. placebo. **(A)** (SB): 4 h (*F* = 6.038; df = 1;11; *p* = 0.049), 6 h (*F* = 7.768; df = 1;11; *p* = 0.0199), 7 h (*F* = 30.995; df = 1;11; *p* = 0.00035). **(B)** (DAB): 6 h (*F* = 28.619; df = 1;15; *p* = 0.0003), 7 h (*F* = 69.161; df = 1;16; *p* = 0.000159).

Administration of FLUD to young animals with SB induced a statistically significant decrease in CORT concentration at 4, 6, and 7 h as compared to the CORT concentration at the same time of day of the same animals in response to placebo administration, that is, basal conditions (Figure 1A). At the same time, the trend toward a decrease in CORT concentration in response to the injection of FLUD in young animals with SB was noted already 3 h after the administration of the FLUD. It looks more vivid when expressing the CORT concentration as a percentage of the initial level: 89 ± 7% vs. 108 ± 20%, respectively, in response to the injection of FLUD and placebo, *p* > 0.05. The lowest concentrations of CORT were observed 7 h after FLUD injection, that is, at 22.00. After 24 h, the concentration of CORT was restored, but has not yet reached the initial values (1128 ± 50 nmol/l and 788 ± 100 nmol/l, respectively, before the injection of FLUD and 24 h after its administration, *p* = 0.017; *F* = 7.177; df = 1;16).

Administration of FLUD to young animals with DAB caused a statistically significant decrease in CORT concentrations compared to the concentration of CORT in response to placebo injection at 6 and 7 h (Figure 1B). The trend toward a decrease in CORT concentration in response to FLUD injection in animals with DAB was noted 4 h after the administration of the FLUD. It looks more vivid when expressing the CORT concentration as a percentage of the initial level: 75 ± 5 and 97 ± 7%, respectively after the administration of FLUD and placebo, *p* > 0.05. After 24 h, the concentration of CORT in response to the injection of FLUD, as in the case of animals with SB, increased, but has not yet reached the initial level. Significantly higher CORT values were found in animals with DAB compared with animals with SB 4 h after administration of FLUD (respectively, 897 ± 70 nmol/l in animals with DAB and 676 ± 60 nmol/l in animals with SB, *p* = 0.037; *F* = 5.26; df = 1;15).

**Figure 2:**
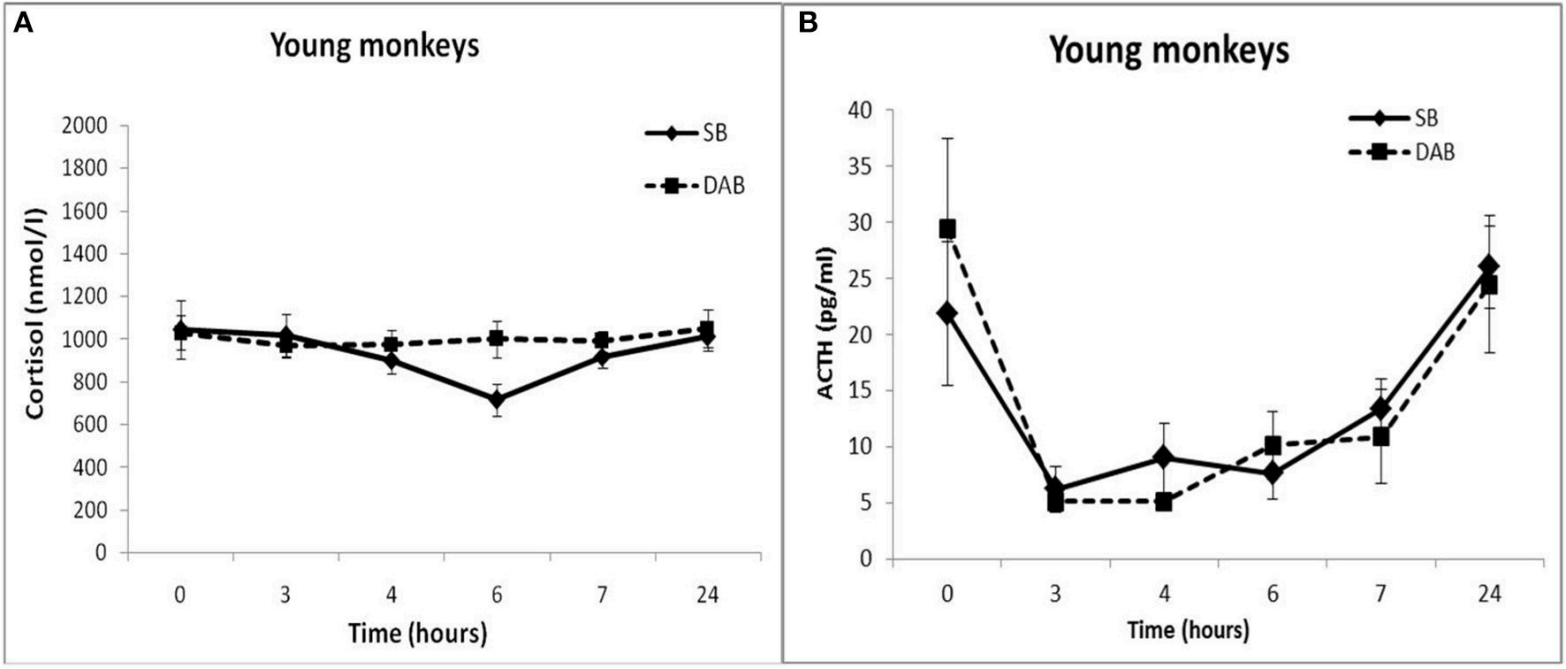
Dynamics of CORT **(A)** and ACTH **(B)** concentrations in peripheral blood plasma of young female rhesus monkeys with SB and DAB in response to administration of placebo (mean ± S.E.M).

Thus, the results of testing the features of the functioning of the MR-dependent negative feedback mechanism in young animals with SB and DAB indicate the development in animals with DAB relative resistance of the HPA axis to the inhibitory effect of FLUD. It's confirmed by the later onset of a decrease in CORT concentration (after 4 h, and not after 3 h, as in animals with SB) and a statistically significantly higher concentration of CORT after 4 h in animals with DAB. The revealed disturbances in the sensitivity of the HPA axis to the feedback regulation mechanism in animals with DAB were not accompanied by statistically significant disorders in the concentration of CORT and ACTH under basal conditions (Figure 2).

#### Old Animals

**Figure 3:**
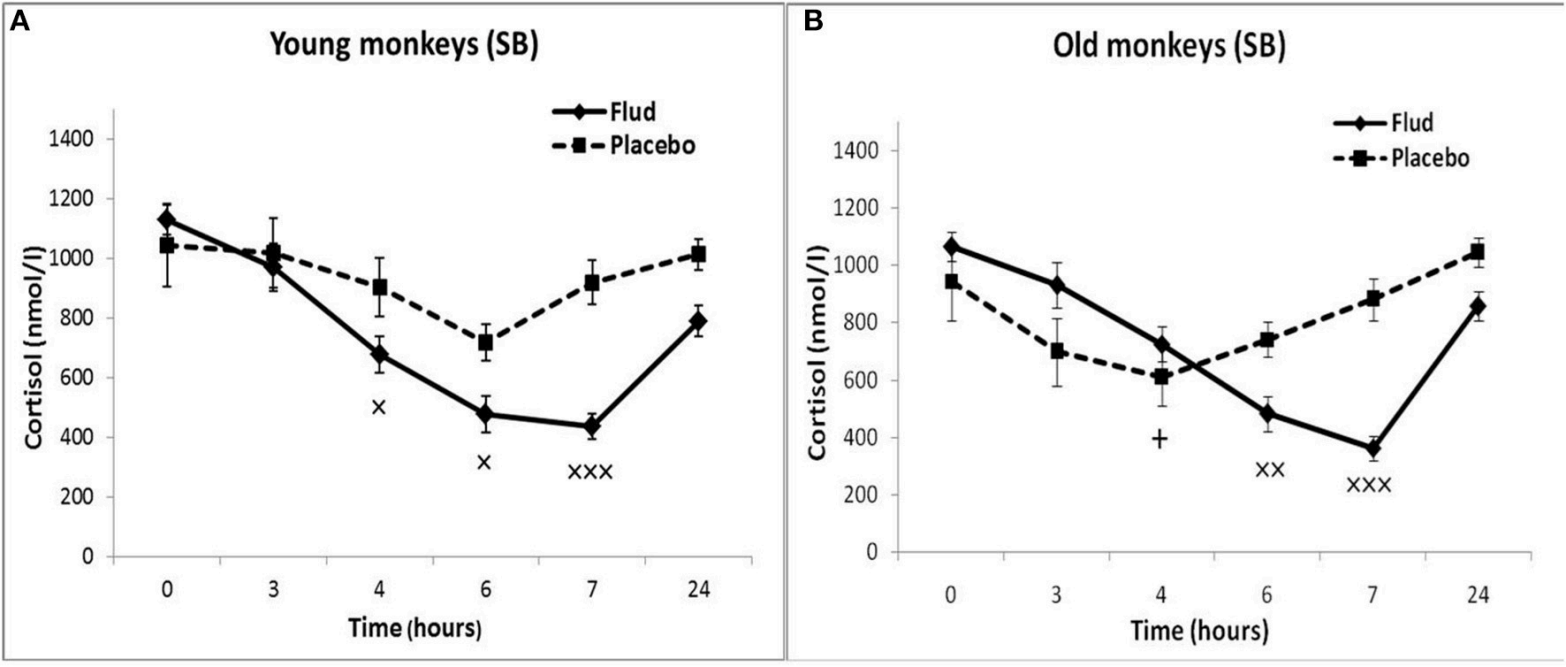
Dynamics of CORT concentration in peripheral blood plasma of young and old female rhesus monkeys with SB **(A)** in response to administration of fludrocortisone and placebo (mean ± S.E.M). +*p* < 0.05—age-related differences (*F* = 7.543; df = 1;10; *p* = 0.029 vs. young animals). ×*p* < 0.05, ××*p* < 0.01, ×××*p* < 0.001 vs. placebo. **(B)** 6 h (*F* = 14.394; df = 1;15; *p* = 0.00467), 7 h (*F* = 30.118; df = 1; 13; *p* = 0.00031).

In response to the administration of FLUD, old female rhesus monkeys with SB demonstrated a significant decrease in CORT concentration at 6 and 7 h compared to the CORT concentration in these animals in response to placebo administration (Figure 3). The minimum values of the CORT concentration in response to the administration of FLUD were after 7 h and had a similar pattern with those in young primates with SB (363 ± 20 nmol/l in old animals vs. 434 ± 50 nmol/l in young animals, *p* > 0.05). However, it should be noted that the decrease in CORT concentration in old animals with SB after the administration of FLUD started later than in young animals with SB (6 h after FLUD injection for old monkeys) compared with the CORT concentration after placebo administration. This resulted in a narrowing of the time range of the FLUD inhibitory effect on the HPA axis function. In other words, these data indicate some decrease in the sensitivity of the negative feedback mechanism at aging in monkeys with SB. However, this decrease in the sensitivity of the HPA axis to the inhibitory effect of FLUD is not accompanied by an increase in CORT levels in response to the administration of placebo, that is, in basal conditions (Figure 3). Moreover, statistically significant age-related differences in CORT concentrations in animals with SB in response to placebo administration with lower values in old animals 4 h after administration of placebo were detected (609 ± 78 nmol/l in old monkeys and 901 ± 60 nmol/l in young animals, *p* = 0.029; *F* = 7.543; df = 1;10). In addition, no significant age-related changes were observed in the basal levels of ACTH in animals with SB (Table 1).

**Table 1:** Dynamics of ACTH concentration in peripheral blood plasma of young and old female rhesus monkeys with SB and DAB in response to administration of placebo (mean ± S.E.M.).

**Group**	**Time, h**					
	**0**	**3**	**4**	**6**	**7**	**24**
**ACTH CONCENTRATION, pg/ml**						
**Young monkeys**						
SB	21.9 ± 6.4	6.2 ± 2.0	9.0 ± 3.1	7.6 ± 2.3	13.4 ± 2.6	26.0 ± 3.7
DAB	29.4 ± 8.0	5.1 ± 0.7	5.1 ± 0.7	10.1 ± 3.0	10.9 ± 4.2	24.4 ± 6.1
**Old monkeys**						
SB	13.8 ± 5.3	7.0 ± 1.7	9.3 ± 1.6	5.8 ± 0.8	10.2 ± 1.9	19.5 ± 2.7
DAB	27.7 ± 9.2	27.7 ± 9.7^*^++	18.0 ± 1.0^*^+++	15.7 ± 1.9^**^	19.7 ± 6.2	27.0 ± 3.0

* *p < 0.05*.

** *p < 0.01 vs. relative values in old animals with SB (3 h: F = 9.112; df = 1;10; p = 0.035; 4 h: F = 13.339; df = 1;10; p = 0.016; 6 h: F = 35.264; df = 1;10; p = 0.002)*.

++ *p < 0.01*,

+++ *p < 0.001 vs. relative values in young animals with DAB (3h: F = 19.092; df = 1;10; p = 0.005; 4 h: F = 91.413; df = 1;10; p = 0.00019)*.

**Figure 4:**
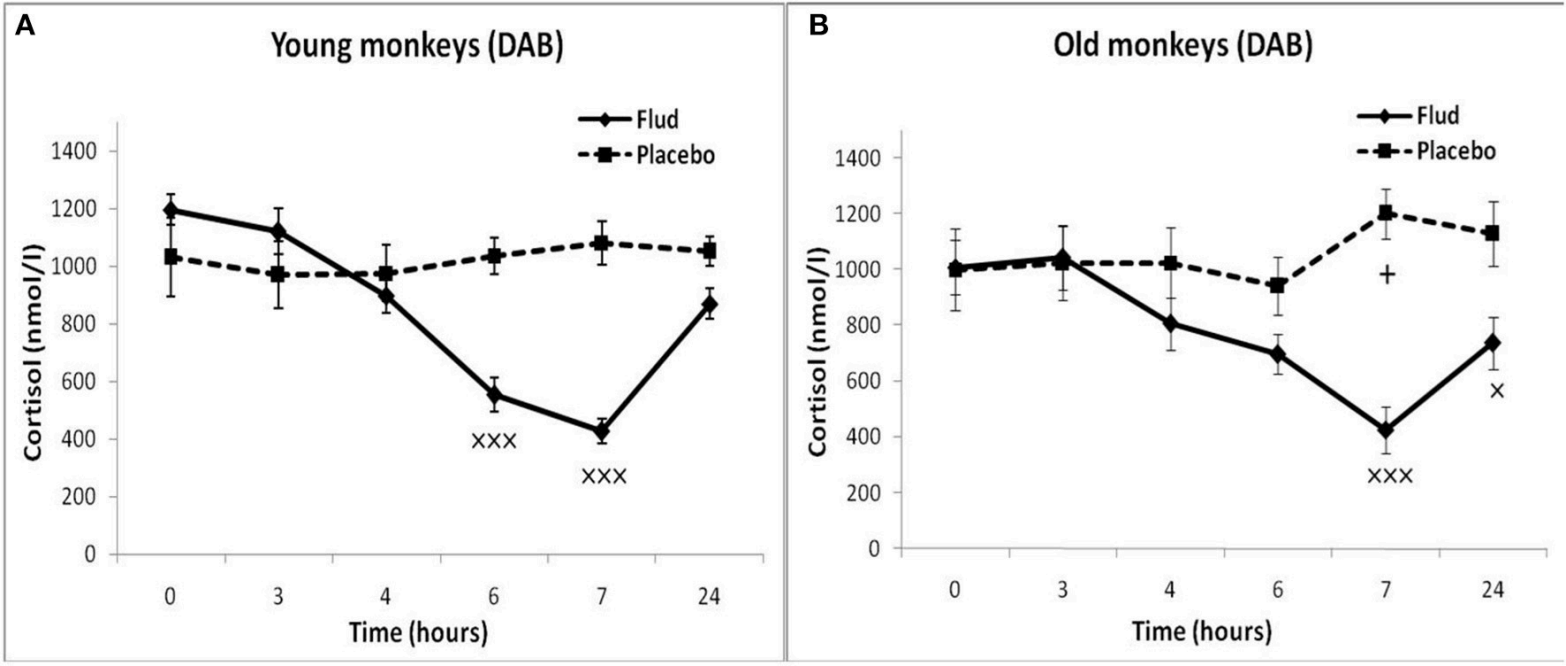
Dynamics of CORT concentration in peripheral blood plasma of young and old female rhesus monkeys with DAB **(A)** in response to administration of fludrocortisone and placebo (mean ± S.E.M). +*p* < 0.05—age-related differences (*F* = 7.363; df = 1;15; *p* = 0.022). ×*p* < 0.05, ×××*p* < 0.001 vs. placebo; **(B)**: 7 h (*F* = 35.414; df = 1;14; *p* = 0.00079), 24 h (*F* = 6.994; df = 1;14; *p* = 0.019).

Old animals with DAB showed statistically significantly lower CORT concentrations in response to the administration of FLUD compared to the values of CORT in response to administration of placebo at 7 and 24 h (Figure 4). This means that a statistically significant decrease in the CORT concentration relative to the concentration of CORT in response to the administration of placebo in the same old animals with DAB occurs later than in young animals with DAB (7 vs. 6 h after FLUD injection relatively for old and young monkeys). In turn, this may be due to some decrease in the sensitivity of the glucocorticoid negative feedback at aging in monkeys with DAB. In addition, we found that CORT concentration in old animals with DAB increased 7 h after placebo administration unlike old animals with SB, in which the reaction of CORT to the placebo administration decreased with aging. Besides, the concentration of ACTH in old animals with DAB was statistically significantly higher than in young animals with DAB 3 and 4 h after placebo administration compared with young animals with DAB (Table 1).

**Figure 5:**
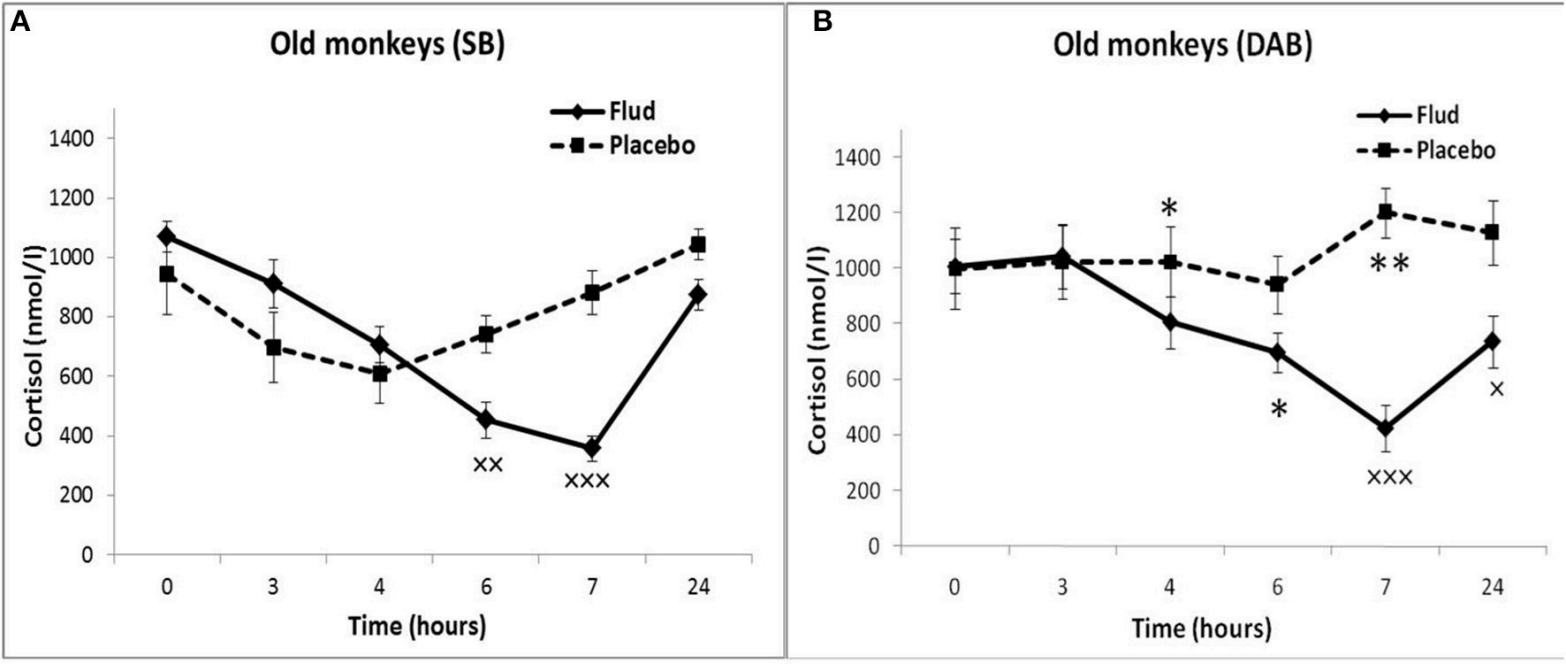
Dynamics of CORT concentration in peripheral blood plasma of old female rhesus monkeys with SB **(A)** and DAB in response to administration of fludrocortisone and placebo (mean ± S.E.M). ×*p* < 0.05, ××*p* < 0.01, ×××*p* < 0.001 vs. placebo; **(B)**: *p < 0.05, **p < 0.01 vs. relative values in animals with SB in response to FLUD or placebo administration (FLUD: *F* = 10.368; df = 1;14; *p* = 0.016; placebo: 4h: *F* = 7.231; df = 1;13; *p* = 0.022; 7 h: *F* = 15.546; df = 1;13; *p* = 0.002917).

In response to the administration of FLUD, old monkeys with DAB showed significantly higher concentrations of CORT than in old animals with SB 6 h after administration of the drug (696 ± 71 nmol/l, respectively, in animals with DAB and 480 ± 30 nmol/l in animals with SB, *p* = 0.016; *F* = 10.368; df = 1;14) (Figure 5). In addition, old animals with DAB exhibited significantly higher concentrations of CORT in relative units after 3 and 6 h after administration of FLUD compared with old animals with SB (103.3 ± 9% vs. 87.5 ± 4% 3 h after FLUD injection, respectively, in animals with DAB and SB: *p* = 0.0482; *F* = 5.088; df = 1;16; 69.0 ± 10% vs. 45.4 ± 3% 6 h after FLUD injection, respectively, in animals with DAB and SB: *p* = 0.0454; *F* = 6.537; df = 1;16**)**.

**Figure 6:**
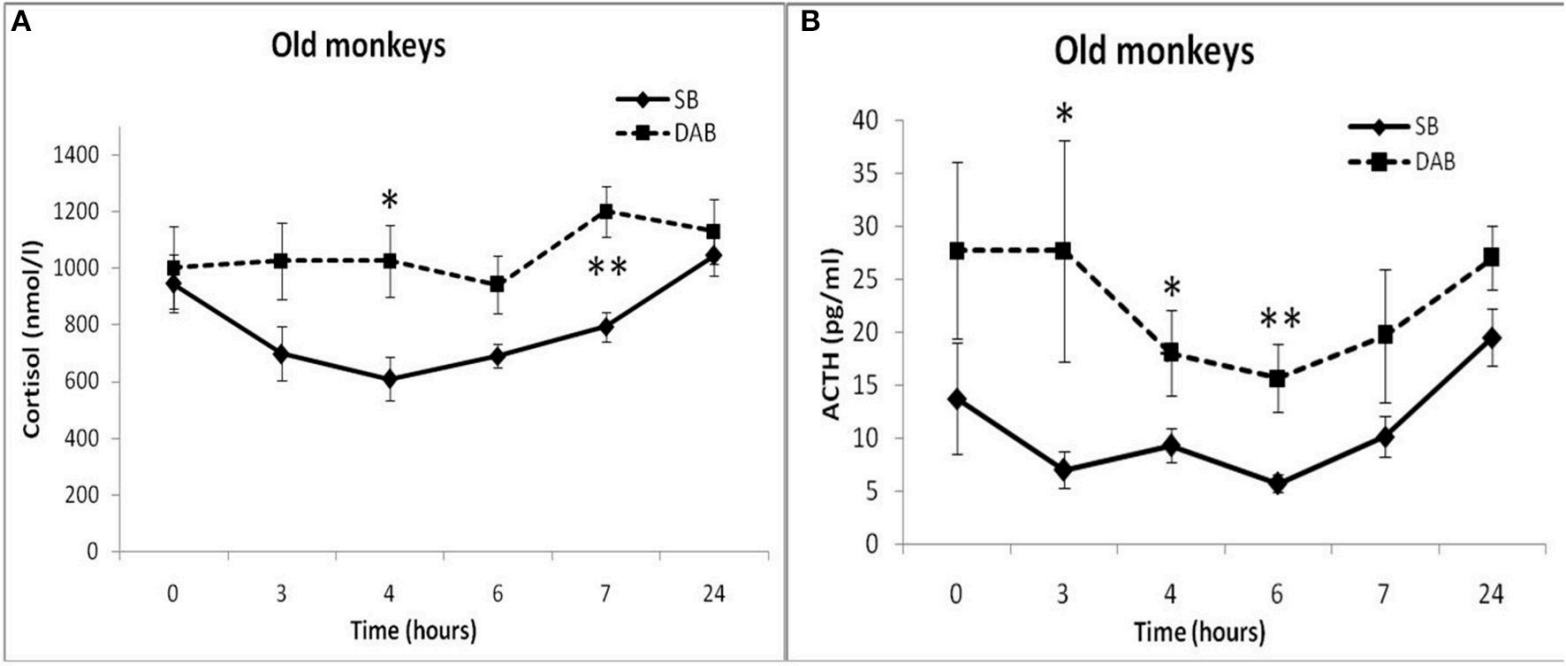
Dynamics of CORT and ACTH concentrations in peripheral blood plasma of old female rhesus monkeys with SB **(A)** and DAB in response to administration of placebo (mean ± S.E.M). *p < 0.05; **p < 0.01 vs. relative values in old animals with SB. **(B)**: 3 h (*F* = 9.112; df = 1;10; *p* = 0.035); 4 h (*F* = 13.339; df = 1;10; *p* = 0.016); 6 h (*F* = 35.264; df = 1;10; *p* = 0.002).

Moreover, old animals with DAB demonstrated higher CORT concentrations 4 and 7 h after placebo administration compared to the corresponding CORT concentrations in old animals with SB (Figures 5, 6). As in the case of CORT levels, old monkeys with DAB showed higher ACTH concentrations 3, 4, and 6 h after placebo administration in old monkeys with DAB compared to old monkeys with SB (Table 1, Figure 6).

The results obtained indicate that during aging in animals with DAB, as in the case of animals with SB, the HPA axis is less sensitive to the inhibitory effect of FLUD, more pronounced in the first h after the drug administration. However, in contrast to animals with SB, age-related disorders in the negative glucocorticoid feedback are accompanied by an increase in basal levels of CORT and ACTH. Different age-related changes in the response of the HPA axis to the test with FLUD in animals with SB and DAB lead to pronounced intergroup differences in the reaction of old animals to FLUD with the development of relative resistance to the glucocorticoid feedback and higher HPA activity under basal conditions in old animals with DAB.

### Studies of the Glucocorticoid Negative Feedback in Regulation of HPA Axis in Young and Old Female Rhesus Monkeys With Various Types of Adaptive Behavior Using Test With Glucocorticoid Receptor

#### Young Animals

The young animals of both groups demonstrated a decrease in CORT concentration in peripheral blood plasma 4, 6, and 24 h after DEX administration. Minimum CORT values were reached 24 h after DEX injection for monkeys with SB, and 6 h after the injection for monkeys with DAB (Table 2). Intergroup differences in the dynamics of the CORT concentration 24 h after DEX administration with higher concentrations of CORT in young animals with DAB were revealed (Table 2). In addition, statistically significantly higher levels of CORT were detected in young animals with DAB compared with young animals with SB 24 and 48 h after administration of DEX when CORT concentration expressed as a percentage of the initial level (Table 3). These data indicate relative resistance of HPA axis to the inhibitory effect of DEX in young animals with DAB compared with young animals of SB.

**Table 2:** Dynamics of CORT concentration in peripheral blood plasma of young and old female rhesus monkeys with SB and DAB in response to administration of DEX (mean ± S.E.M.).

**Group**	**Time, h**				
	**0**	**4**	**6**	**24**	**48**
**CORTISOL CONCENTRATION, nmol/l**					
**Young monkeys**					
SB	1220 ± 31	698 ± 59###	452 ± 52###	371 ± 50###	1038 ± 31##
DAB	1104 ± 41	670 ± 43###	527 ± 84###	691 ± 110#^*^	1064 ± 35
**Old monkeys**					
SB	1011 ± 78**+**	667 ± 42##	516 ± 50###	259 ± 58###	834 ± 92
DAB	988 ± 43	646 ± 35##	482 ± 54###	228 ± 61###**+**	667 ± 116

* *p < 0.05 vs. relative values in young animals with SB (F = 6.953; df = 1; 9; p = 0.0327)*.

+ *p < 0.05 vs. young animals with DAB (0 h: F = 5.400; df = 1;11; p = 0.047; 24 h: F = 11.341; df = 1;9; p = 0.0106)*.

# p < 0.05;

## p < 0.01;

### *p < 0.001 vs. the initial values (0 h)*.

*SB, young: 4 h (F = 61.722; df = 1;10; p = 0.000194); 6 h (F = 162.539; df = 1;10; p = 0.000187); 24 h (F = 191.891; df = 1;10; p = 0.000187); 48 h (F = 17.653; df = 1;10; p = 0.00197). SB, old: 4 h (F = 15.039; df = 1;12; p = 0.00235); 6 h (F = 28.377; df = 1;12; p = 0.00034); 24 h (F = 59.799; df = 1;12; p = 0.00017)*.

*DAB, young: 4 h (F = 52.703; df = 1;8; p = 0.00028); 6 h (F = 38.137; df = 1;8; p = 0.00045); 24 h (F = 11.017; df = 1;8; p = 0.011). DAB, old: 4 h (F = 20.922; df = 1;10; p = 0.0012); 6 h (F = 29.594; df = 1;10; p = 0.00044); 24 h (F = 59.924; df = 1;10; p = 0.00019)*.

**Table 3:** Dynamics of CORT concentration in peripheral blood plasma of young and old female rhesus monkeys with SB and DAB in response to administration of DEX as a percentage of baseline (mean ± S.E.M.).

**Group**	**Time, h**				
	**0**	**4**	**6**	**24**	**48**
**CORTISOL, %**					
**Young monkeys**					
SB	100 ± 0	57.2 ± 4.1	37.0 ± 3.9	30.4 ± 4.2	85.1 ± 0.8
DAB	100 ± 0	60.7 ± 4.3	47.8 ± 7.6	62.6 ± 9.1^*^	96.4 ± 2.8^**^
**Old monkeys**					
SB	100 ± 0	65.9 ± 6.9	51.0 ± 8.7	25.6 ± 6.3	82.4 ± 7.6
DAB	100 ± 0	74.4 ± 6.1	48.8 ± 8.4	23.1 ± 7.9**+**	67.5 ± 17.9

* *p < 0.05*,

** *p < 0.01 vs. relative values in young animals with SB (24 h: F = 11.116; df = 1;9; p = 0.011; 48 h: F = 21.854; df = 1;9; p = 0.002)*.

+ *p < 0.05 vs. relative values in young animals with DAB (F = 10.399; df = 1; 9; p = 0.013)*.

#### Old Animals

The dynamics of CORT concentration in old and young female rhesus monkeys with SB in response to the administration of DEX was of a similar character, that is, it decreased 4, 6, and 24 h and approximately restored 48 h after the administration of DEX (Table 2). However, the concentration of CORT in old animals with SB 6 h after the DEX administration showed a tendency to higher values compared with young animals of similar behavior in absolute (Table 2) and relative units (Table 3).

Unlike animals with SB, old animals with DAB showed significantly lower CORT concentrations (both in absolute and in relative units) compared to young animals with DAB 24 h after the DEX administration and a tendency to lower values 48 h after the administration of DEX (Tables 2, 3). Moreover, CORT concentrations in old animals with DAB did not return to baseline values, unlike young animals with DAB and animals with SB from both age groups. In contrast to young animals of various behavioral groups, there were no differences in the reaction of HPA axis to the administration of DEX in old animals with SB and DAB (Tables 2, 3).

These findings suggest both no significant age-related changes in the sensitivity of HPA axis to the DEX inhibitory effect in animals with SB, and the increased sensitivity of HPA axis to DEX in animals with DAB. In turn, the increase in the sensitivity of HPA axis to DEX at aging in monkeys with DAB is apparently the main reason for the lack of intergroup differences in HPA axis sensitivity to DEX in old monkeys, the presence of which was noted in young animals of similar behavioral groups.

## Discussion

### Features of the Glucocorticoid Negative Feedback in Regulation of HPA Axis Based on Mineralcorticoid Receptor in Young and Old Female Rhesus Monkeys With Various Types of Adaptive Behavior

#### Young Animals

The revealed inhibitory effect of FLUD on HPA axis activity in all young animals, regardless of their behavior, testify the important role of MR in self-regulation of HPA axis in young primates in the evening, then there is a period of physiological circadian nadir of basal activity of HPA axis. The pronounced inhibitory effect of FLUD on HPA axis activity during the phase of circadian decline in its activity was also observed in young healthy people (22, 37–39). In addition, pharmacological doses of FLUD could significantly reduce the stimulating effect of metyrapone on ACTH and 11-deoxycorticosterone levels in the blood of young individuals (37). There is also a publication that various FLUD doses were able to decrease the HPA axis activity stimulated by the CRH administration during the nadir phase of its circadian rhythm. This, thereby, emphasizes the role of MR in the CRH-stimulated activity of HPA axis (24).

At the same time, the results of the FLUD test indicate the presence of intergroup differences in the glucocorticoid feedback in young animals under MR activity stimulation. Animals with DAB demonstrated relative resistance of HPA axis to the inhibitory effect of FLUD in the early exposure period−3 and 4 h after FLUD injection. However, these changes did not lead to significant disturbances in the functioning of HPA axis in animals with DAB under basal conditions. So, the concentration, as CORT, and ACTH did not undergo significant intergroup differences in response to placebo administration.

One possible explanation for the identified intergroup differences in CORT levels in young animals could be lower concentrations of MR in the hippocampus in young monkeys with DAB. As shown by numerous data, the hippocampal MR perform a negative feedback control of HPA axis activity (4, 24, 25, 40). Apparently, at a young age in primates with DAB, the MR concentration in the hippocampus is slightly lower than in young animals with SB. This assumption is consistent with a number of literature data. So, in pathoanatomical material from patients with depression, including young age, it was revealed that the expression of mRNA for MR in the hippocampus is essentially lower compared to people without depression (41). In addition, it has been shown that stimulation of MR with FLUD decreases the secretion of CORT and improves cognitive function in individuals with depression (22).

#### Old Animals

The decrease in CORT concentration in old animals with SB after the administration of FLUD started later than in young animals with SB compared with the CORT concentration after placebo administration (6 h for old monkeys vs. 3 h for young ones). These data indicate some decrease in the sensitivity of the glucocorticoid negative feedback in regulation of the HPA axis at aging in animals with SB. Besides, it should be noted that there are age-related differences in the dynamics of CORT in response to placebo administration, which are apparently due to age-related disturbances in the circadian rhythm of CORT secretion. So, the minimum CORT values in young animals with SB were observed 6 h after the placebo administration, while they were registered earlier (in 4 h) in old animals with SB. An earlier and significantly lower circadian decrease in CORT levels in old animals with SB seems to have caused a later decrease in their CORT concentrations in response to the administration of FLUD. This later decrease in CORT concentrations results in a narrowing of the time range of the inhibitory effect of FLUD on HPA axis function.

With aging, animals with DAB demonstrated a later decrease in CORT secretion in response to the administration of FLUD compared with the CORT concentration after placebo injection then in young animals with DAB (7 h for old monkeys vs. 6 h for young ones). These results indicate that during aging in animals with DAB, as in the case of animals with SB, the HPA axis is less sensitive to the inhibitory effect of FLUD. However, in contrast to animals with SB, age-related disorders in the negative glucocorticoid feedback are accompanied by an increase in basal levels of CORT and ACTH. Different age-related changes in the response of the HPA axis to the test with FLUD in animals with SB and DAB lead to pronounced intergroup differences in the reaction of old animals to FLUD. They were associated with the development of relative resistance in the glucocorticoid feedback and higher HPA activity under basal conditions in old animals with DAB (see Figures 4–6).

Information about the differences in age-related disturbances of the glucocorticoid feedback based on MR in individuals differing in their psychophysiological status is extremely rare. Nevertheless, the available array of literature data on the features of age-related disorders in HPA regulation by the feedback mechanism, as well as HPA axis activity under basal conditions and under stress, indicates the ambiguity of the data obtained. A number of clinical studies have demonstrated an increase in basal concentrations of ACTH and CORT associated with aging during the quiescent phase nadir of HPA axis circadian rhythm (42–45). In addition, it was shown that the acute administration of agonists (FLUD) and antagonists (canrenoate) of MR in the evening, that is, during the circadian phase of the decrease in HPA axis activity, results in a lower decrease (38) or an increase (45) activity of the HPA axis in old subjects compared to young subjects. This may reflect age-related damage to MR function. Age-related damage to the hippocampal MR function was recorded not only in humans, but also in laboratory animals (46–49). At the same time, the association of the age-related reduction of MR with reduced inhibition of PVN activity in the hypothalamus (48, 49) and an increase in the amount of CRH in the pituitary portal venous system and the concentrations of ACTH and corticosterone in the general circulation (50–52) were identified.

At the same time, there are papers that showed no pronounced disturbances in the MR function when aging in humans or animals. Thus, some studies have demonstrated a lack of distinguishable effect of spironolactone on the basal secretion of the HPA axis or on the response of the HPA axis to the CRH administration during the nadir phase of the CORT secretion circadian rhythm (53–55). Perhaps, the inconsistency of the literature data is due not only to the use of different doses of drugs and different experimental models, but also due to the lack of an individual approach to the object of research, in particular, based on differences in the characteristics of adaptive behavior, on differences in traits of anxiety, on psycho physiological indicators.

In the present study, we demonstrated for the first time the results of a test with the FLUD (MR agonist), which indicate differences in the feedback regulation of the HPA axis during aging in rhesus monkeys that differ in the characteristics of higher nervous activity (mild/moderate stress behavior). More pronounced age-related disorders in the glucocorticoid negative feedback developed in old animals with DAB than in old animals with SB. Old primates with DAB showed signs of relative refractoriness of the HPA axis to regulation by feedback mechanism compared to old SB animals. The revealed age and intergroup differences in the feedback regulation of the HPA axis in primates with SB and DAB led to the formation of pronounced intergroup differences in the activity of the HPA axis in basal conditions in the evening with higher activity in old animals with DAB. The relationship in the feedback regulation disorders of the HPA axis and MR levels in the hippocampus with the psychophysiological characteristics of individuals was also noted in the literature, with emphasis on individuals with depression and anxiety (41, 56, 57). It was also found that as a result of antidepressant therapy of patients with depression, the level of hippocampal MR increases (41, 56, 58). At the same time, the administration of spironolactone (MR antagonist) induces a greater increase in CORT levels in patients with major depression than in the control group (59). All these data give grounds to believe that the MR function is involved not only in the pathophysiology of depression, but, as our data showed, in the dysfunction of the HPA axis in old primates with DAB in basal conditions and under stress.

### Features of the Glucocorticoid Negative Feedback in Regulation of HPA Axis Based on Glucocorticoid Receptor in Young and Old Female Rhesus Monkeys With Various Types of Adaptive Behavior

#### Young Animals

In addition to differences in the functioning of the HPA axis in SB and DAB primates detected in the FLUD test, our experiments also revealed a number of pronounced intergroup and age-related differences in the response of the HPA axis to the administration of DEX. Thus, young animals with DAB showed a relative resistance of CORT secretion to the inhibitory effect of DEX 24 h after its administration compared to young animals with SB. In addition, in relative units, statistically significantly lower concentrations of CORT also were recorded in young animals with DAB 48 h after the administration of DEX. These data are in good agreement with the literature data demonstrating the relative resistance of the HPA axis to the suppression of DEX in people with depression (60–64). Deficiency of the GR function may underlie the relative resistance of the HPA axis to the suppression by DEX. Indeed, studies on small laboratory animals showed that administration of DEX did not lead to a reduction in corticosterone levels in heterozygous, GR knockout mice to the same level as in free-living mice (65). It is interesting that the administration of DEX into different regions of the young rat brain led to a reduction in glucocorticoid levels in the general circulation. This is apparently due to the wide spread of GR in the brain and indicates that different parts of the brain (in particular, the PVN of the hypothalamus, hippocampus and prefrontal cortex) participate in the regulation of the HPA axis by the mechanism of negative feedback (66).

#### Old Animals

Our data indicate a different direction of the age-related changes in the feedback regulation of the HPA axis on the basis of GR in primates with SB and DAB, namely, a tendency to decrease in sensitivity in animals with SB and, conversely, an increase in sensitivity in animals with DAB. These differences, apparently, are due to the fact that the number of GR in animals with SB decreases slightly while one increases in animals of DAB. The data on development during aging the tendency to relative resistance of the HPA axis to the GR-based feedback regulation in animals with SB are in good agreement with our previous studies. Earlier, our investigations showed the resistance of the feedback mechanism in the regulation of CORT secretion during aging in female rhesus monkeys without subdividing them into groups, depending on the behavior (67–69). In addition, our data are consistent with results of other authors in primates (70), humans, and rodents (48, 49, 71). Thus, in an experiment on rodents with aging, there was a decrease in the number of GR in the hippocampus with reduced inhibition of the PVN hypothalamus (48, 49, 71). It was also found that there was no pronounced suppressive effect of DEX in old rats compared to young rats when it was administered into various regions of the brain. It indicates the development in old animals of HPA axis resistance to regulation by the negative feedback at the level of the brain (66).

At the same time, our data on increasing sensitivity of the HPA axis to the inhibitory effect of DEX during aging in primates with DAB are consistent with the results of a number of clinical studies (63, 72). Earlier it was revealed that the hyper suppression of CORT levels in the DEX test in women with a history of stress in the early period of life (63, 72). It should be emphasized (see the section Materials and Methods) that two of our experimental animals with DAB underwent maternal deprivation in the first 1.0–9.0 months after birth, that is, a pronounced stress effect in the early period of life. In addition, earlier we published data that the vast majority of adult and old female rhesus monkeys subjected to maternal deprivation in the early postnatal period show anxiety-like or depression-like behavior, as well as damage in the functioning of the HPA axis (73). Moreover, there is a definite correlation between our previously published studies (13, 14, 19) with the results of works (63, 74, 75), who demonstrated that women with a history of stress in the early period of life and without depression at the time of the survey demonstrate increased ACTH response to stress and CRH administration.

The observed disturbances in the sensitivity of young and old primates from DAB to DEX suppression (in any case, in individuals undergoing maternal deprivation) may be due to epigenetic damage to GR expression. Thus, a number of studies have shown that GR in humans and animals are sensitive to epigenetic changes in response to experiments in the early period of life (76–79). In particular, a recent study demonstrated that the expression of the GR gene (*Nr3c1*) in various nuclei of the hypothalamus (in particular, in PVN) and the GR regulator (FKBP51) in old mice representing the progeny of females with diet -induced obesity, increased in basal conditions, and in response to stress exposure (79). It is known that the genetic polymorphism of the gene *Nr3c1* GR and the protein regulating GR, the so-called FK506-binding protein 51 (*Fkbp5* gene), is associated with the pathophysiology of mood disorders in the human population (80).

On the other hand, tendency to increasing sensitivity of the HPA axis in old animals with DAB to the inhibitory effect of DEX may be caused by a disruption in the circadian rhythm of GR concentration in brain structures involved in the regulation of the HPA axis by the negative feedback such as the hippocampus, PVN, anterior pituitary, and etc. We have previously demonstrated that the sensitivity of the feedback mechanism in the regulation of CORT secretion, determined by the DEX test, depends on the time of day, age, and is of an individual character associated with the characteristics of adaptive behavior (81). In particular, it has been demonstrated that the sensitivity of the HPA axis to the inhibitory effect of DEX in old animals with DAB, regardless of the time of administration of DEX, is increased compared to young animals with DAB. In the literature there is information on the increase in the number of non-nuclear GR with aging in medial prefrontal cortical glial subpopulations (82).

Thus, the data obtained by us testify to pronounced individual differences in the sensitivity of the glucocorticoid feedback based both on MR and on GR, in female rhesus monkeys. In animals with increased anxiety (DAB groups), the glucocorticoid negative feedback largely differs from the analogous mechanism in animals with SB at a young age and even more with aging and, apparently, plays a big role in pathophysiology of individual and age-related disorders of HPA axis stress response, previously identified (13, 14, 20). Disturbances in the ratio of MR and GR in brain structures appear to underlie intergroup and age-related disorders in the regulation of HPA activity by the negative feedback. Perhaps, in young animals with DAB, there is a deficiency of MR and GR. While aging of animals with SB (physiological aging) presumably leads to a small deficiency of MR and, apparently, GR, tests with FLUD, and DEX in old monkeys with DAB may indicate the development of pronounced deficiency of MR activity and, possibly, increased GR activity. Significant dysfunction of MR and GR in old animals with DAB, perhaps, leads to a sharp disruption in the ratio MR: GR in different structures of the brain and, in particular, the hippocampus. In turn, because MR and GR not only regulate the activity of HPA axis, but also actively participate in cognitive processes (30–33, 80), the assumed pronounced disturbances in the ratio of MR: GR in primates with DAB can contribute to intergroup and age differences in the cognitive abilities of primates, as well as post stress syndrome, psychiatric and possibly neurodegenerative diseases.

This conclusion is in good agreement with the results of experiments that demonstrated differences in the effects of GR and MR activation on the survival and death of hippocampal neurons. While GR activation by high levels of glucocorticoids promotes neuronal death by stopping the cell cycle and inducing apoptosis (83, 84), activation of MR is involved in the survival of the hippocampal neurons, in cognitive functions, in the integrity and stability of neuronal networks (26, 85, 86). In addition, forebrain MR over expression enhanced memory, reduced anxiety-like behavior, attenuated neuronal loss in cerebral ischemia (87), and attenuated HPA axis response to stress in mice (88). At the same time, MR antagonists increased HPA axis activity in humans (89). At the same time, prolonged administration of tricyclic antidepressants improved cognitive damage in old rats due to upregulation of MR in parallel with a lower secretion of corticosterone (90).

Overall, our results testify the pronounced individual and age differences in the negative feedback regulation of HPA axis, presumably resulting from unequal individual, and age-related changes in the activity of MR and GR in the brain structures supporting the functions of the HPA axis. The maximum age disorders in the functioning of the negative feedback regulation of HPA axis with a pronounced decreased sensitivity to fludrocortisone are characteristic of animals with DAB, which underlie increased HPA axis activity in the basal conditions and, apparently, the increased vulnerability of these animals to stress exposure, revealed by us earlier.

## Author Contributions

NG designed the study, supervised the work, performed the experiments, analyzed the results, and wrote the paper. OC performed the experiments, conducted statistical analyses. NR performed the experiments, measured concentrations of hormones, and conducted statistical analyses. TO performed the experiments and measured concentrations of hormones. All authors have approved the final manuscript.

### Conflict of Interest Statement

The authors declare that the research was conducted in the absence of any commercial or financial relationships that could be construed as a potential conflict of interest.
